# Aplasia cutis congenita: a report of two cases from National Hospital Abuja, Nigeria and review of the literature

**DOI:** 10.11604/pamj.2020.36.291.24523

**Published:** 2020-08-17

**Authors:** Mariya Mukhtar-Yola, Lauretta Mshelia, Amsa Baba Mairami, Adekunle Tolutope Otuneye, Edith Terna Yawe, Patricia Igoche, Lamidi Isah Audu

**Affiliations:** 1 Department of Paediatrics, National Hospital Abuja, Abuja, Nigeria,; 2 Neonatology Division, National Hospital Abuja, Abuja, Nigeria

**Keywords:** Aplasia cutis congenita, scalp, newborn, Nigeria

## Abstract

Aplasia cutis congenita is a rare congenital abnormality first described in 1767 by cordon. It mostly appears as a solitary lesion involving various layers of the skin and sometimes the bone on the scalp, limbs or abdomen. Genetics, environmental and exogenous causes have been implicated as potential causes. Only about 500 cases have been reported globally as of 2013. Two cases of Aplasia Cutis Congenita (ACC) who presented with scalp and bone defects at birth are reported, one in a syndromic child delivered to a consanguineous family, with associated cardiac, skin and nail anomalies (likely Adams Oliver syndrome) and the other as an isolated scalp lesion. Both were large defects managed conservatively by a multidisciplinary team. The challenges of investigating and managing such complex scalp anomalies in sub-Saharan Africa are highlighted.

## Introduction

Skull and skin defects are rare and devastating malformations to parents and clinicians alike. Skin defects at birth are termed Aplasia Cutis Congenita (ACC); first described by Cordon in 1767 in two sisters with skin defects of the lower extremities [1]. It is a rare congenital abnormality involving variable layers of the skin, mostly seen as solitary lesions involving the midline over the skull vertex and, less commonly underlying periosteum and bone [2]. It has also been reported in other sites such as the chest, abdomen and limbs [3]. The National Organization for Rare Disorders (NORD) has described other synonyms for ACC as: congenital defect of the skull and scalp, congenital ulcer of the newborn and scalp defect congenital [4]. The etiology and pathophysiology of ACC are not well understood, rather it appears to be multifactorial in some, as both genetics, environmental and exogenous causes have been implicated [5, 6]. Autosomal dominant, autosomal recessive as well as sporadic cases have been reported [7-9]. The most widely accepted pathophysiology model describes the tension that disrupts the skin from properly approximating during fetal development [10]. This can be from factors like poor blood supply to the skin, fetal and placental ischemia, intrauterine infections and failure in neural tube closure [10, 11].

Rupture of amniotic membrane in early pregnancy forming amniotic bands [5], has also been postulated to be a possible cause. Suspected exogenous causes include teratogenic substances like methimazole, carbimazole, misoprostol, valproic acid and trauma [6]. Until recently, no specific genetic target had been identified, but a recent study showed the BMS1 gene plays a possible role [8]. Aplasia cutis congenita can also be associated with several genetic syndromes, including Adams-Oliver syndrome, Bart syndrome, Setleis and Patau syndromes [6]. The incidence of ACC is approximately 1 to 3 out of 10,000 births [3, 12] with case fatality rate as high as 18-50% [7, 10]. There is no significant gender or cultural predilections reported in literature and the lesions are typically noticed at birth, although parents may not seek care for several months especially if lesions are small [10]. The lesions may also heal in utero, with scars noticed at birth [10]. It is a rare condition as only about 500 cases have been reported globally as of 2013 [11, 13] and these were all outside Africa. Although, the membranous type of aplasia cutis congenita is most common, the defect may involve only the epidermis and dermis, resulting in mild scarring or it may extend into the subcutaneous tissue or rarely periosteum, skull and the dura [10].

**Historical perspective:** although, Cordon was the first to describe this condition in 1767 [1], it was William Campbell that first published a case report in 1826 of two siblings, who had scalp skin defects unfortunately, both died, one from haemorrhage and the other from hydrocephalus [13]. Since then several reports have emerged describing lesions involving extremities, shilling size scalp lesions, partially healed lesions and ACC associated with congenital malformations. More recently, Terruhn in 1930 conducted a review involving 108 patients that showed involvement of the scalp in 79 cases and other sites in 29 cases [14]. Maillet-Declerck *et al*. reported their experience with 29 cases managed between 1976-2011 with defect size ranging from 1-192 cm [15]. Thirteen of these patients had bone aplasia, 5 were managed conservatively, while 24 had various forms of surgery. Four died in the newborn period from infections while the others achieved complete healing [15]. From Central Africa, a case with a scalp defect was reported from Rwanda [16]. Recently in Nigeria, a few case reports have been published from the north central and southern part of the country [13, 17, 18], all however involved the limbs. To the author´s knowledge, this is the first report from Nigeria involving scalp lesions.

## Patient and observation

**Figure 1:**
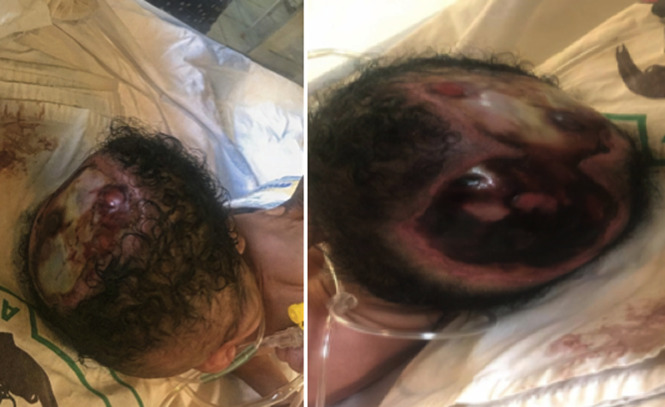
case 1 at admission

**Figure 2:**
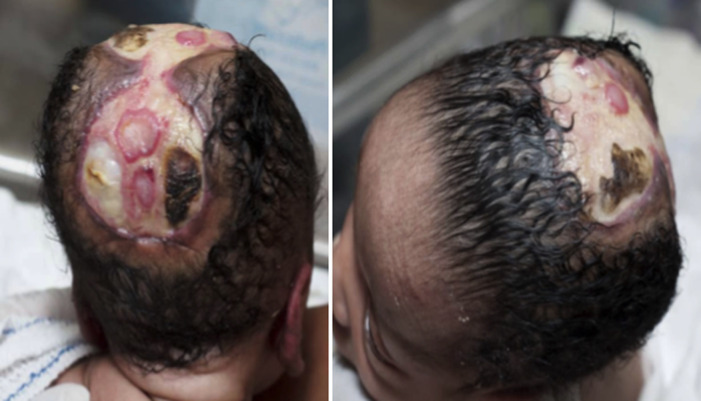
case 1 at 14 days of admission

**Case 1:** baby NU, a male infant from Gbayi tribe delivered at term in a hospital by spontaneous vaginal delivery to a 30-year-old para 2 mother. Baby cried well at birth and weighed 3kg. He was referred at age of 4 hours on account of absence of skin and skull bone noticed at birth. Pregnancy was supervised and uneventful and mother denied use of any medications orthodox or herbal except for antenatal drugs. Ultrasound scan in second trimester was reported as normal. He was the third child of parents in a monogamous consanguineous family setting parents being cousins. Though there was a history of a fresh still birth during the mother´s first confinement, no congenital malformations were seen in that baby and there was no history of congenital anomalies in the extended family. Mother is a 30-year-old teacher and father a 35-year-old police officer and both had a secondary level of education. Physical examination revealed an irregularly shaped well-demarcated large defect on the vertex of the scalp over the parietal bone bilaterally extending to the occipital region measuring 10 x 8 cm in the midline and 6 x 6 cm in the occipital region. A yellow layer of membrane covered the defect. The patient had low set ears, he was irritable, with a tense anterior fontanelle and in respiratory distress. Other system examination were unremarkable. A diagnosis of aplasia cutis congenita with possible meningitis was made (Figure 1 and Figure 2). Baby had routine care as per protocol including: oxygen, intravenous fluids, antibiotics at meningitis doses, dressing with sofra-tulle and normal saline soaked gauze.

**Figure 3:**
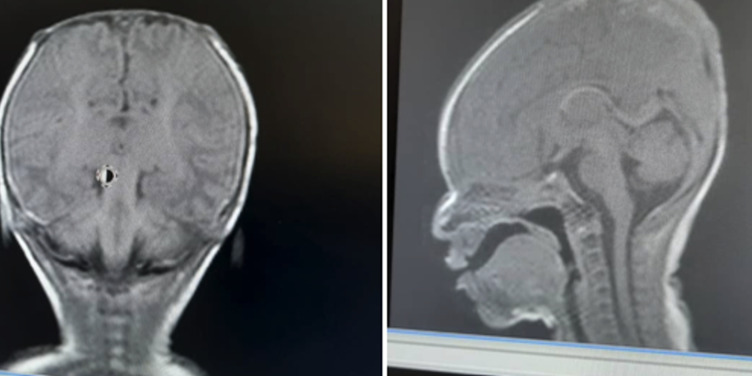
brain MRI for case 1

The respiratory distress subsided; oral feeds were eventually commenced and well tolerated. Multidisciplinary team management was done along with neurosurgeons and plastic surgeons. A babygram and an abdominopelvic scan done were normal. The brain magnetic resonance imaging (MRI) showed absence of parietal bone towards the vertex bilaterally extending from 2 cm behind the anterior fontanelle down to the posterior fontanelle giving an area of defect measuring 8.3 cm x 5.3 cm (AP X TR). The subcutaneous scalp tissues were not visualized. However, the brain in this region was overlaid by the dural meninges. The occipital, frontal and temporal bones of the cranial vault and their overlying scalp tissues were spared. The cerebral hemispheres, cerebellum and the brain stem structures were normal in morphology and signal intensity. No intra-axial or extra-axial mass or collection seen. No area of abnormal enhancement seen. The cortical sulci, sylvian fissures, basal cisterns and ventricles are normal in calibre, outline and intensity. The orbits and retro-orbital structures are within normal limit. Conclusion: features in keeping with aplasia cutis congenita (Figure 3). Burns and plastic unit and neurosurgeons made plans for future intervention in form of raising a flap cover for autologous skin grafting. Baby was discharged home after 18 days of admission on oral antibiotics and daily wound dressing, for follow up at the specialty clinics.

**Case 2:** baby YA, Hausa by tribe was referred at 48 hours of life on account of absence of part of the skull bone noticed at birth. Baby was delivered per vaginum at home, delivery was not associated with overt trauma to the head. Patient was a product of term gestation; birth weight was unknown though admission weight was 2 kg. Mother was 25-year-old and not gainfully employed, while father was a hospital attendant. Baby was the 3^rd^ child of parents in a monogamous, non-consanguineous setting; the two older siblings were alive and well. No known family history of congenital anomaly. There was no history of use of herbal medications and mother was not on any other medication. There was no history of exposure to radiation in pregnancy and parents did not live close to a network mask. Mother had no obstetric scan done in pregnancy. Important clinical findings at presentation were a very ill patient, febrile, cyanosed, hypertonic with a midline subcutaneous tissue defect of the scalp extending from the frontal to the occipital area extending to the right parietal region.

**Figure 4:**
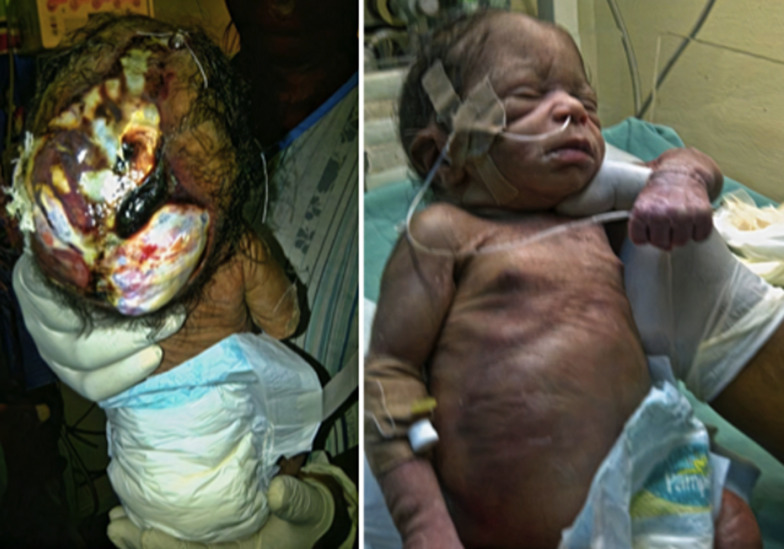
case 2 at admission

There was leakage of cerebrospinal fluid (CSF) from a likely dural involvement. He had desquamating skin, hypoplastic finger and toe nails, visible dilated abdominal wall veins as well as a grade 3 systolic cardiac murmur loudest at the left lower sternal edge. Other systems were essentially normal. A working diagnosis of multiple congenital anomalies (congenital cutis aplasia and congenital heart disease (ventricular septal defect) with meningitis was made. Differentials of Adams-Oliver syndrome and Patau´s syndrome were considered (Figure 4). Sepsis screen yielded *Providencia specie* and *E. coli* from blood and CSF respectively. Abdominal ultrasound scan was normal, unfortunately, brain MRI, echocardiography and chromosomal studies could not be done due to financial constraints. He was treated with parenteral antibiotics and wound was cleaned daily and dressed with betadine. Complications included CSF leakage and meningitis, which resolved over time. The burns and plastic unit planned for prolene mesh/titanium mesh and latissimus dorsi musculocutaneous flap cover subsequently. Patient was discharged to parents on request after 35 days on admission, however baby died 7 days post discharge.

## Discussion

Aplasia cutis congenita is primarily a clinical diagnosis with no specific histological alterations [2]. We have reported two cases of ACC presenting with scalp defects at birth, one in a syndromic child and the other as an isolated lesion. In both babies the skin defects were large sheets, irregular and involved hypoplasia of the subcutaneous tissues including the bones. Frieden and Schierz reported that 70% to 90% of lesions are localized to the vertex of the scalp presenting as solitary or multiple lesions that are non inflammatory, well demarcated, sheet-like, or appear as patches with irregular shapes, size and involving variable skin depths [19, 20]. Others have reported that lesions may be circular, oval, linear or stellate in configuration [10]. The proximity of scalp aplasia cutis congenita to the scalp hair whorl, which is thought to be the point of maximum tensile force during rapid brain growth, has led to the hypothesis that tension induced disruption of the overlying skin occurs at 10-15 weeks of gestation when hair direction, patterning and rapid brain growth occur. This may also explain the increased incidence of aplasia cutis congenita on the vertex scalp [10]. At birth, the lesions may already be healed with scarring or may remain superficially eroded or deeply ulcerated, occasionally involving the dura or the meninges [10]. In the cases presented, both defects were deeply eroded and in one case involved the dura leading to CSF leakage and meningitis. In 1986, Frieden proposed a classification system for ACC that included nine groups based on characteristics of the lesion, associated abnormalities and type of inheritance [19].

Group one encompasses Scalp ACC without multiple anomalies and can be inherited autosomal dominantly or sporadically. Group two consists of scalp ACC with associated limb abnormalities (limb reduction abnormalities; syndactyly; club-foot; nail absence or dystrophy; skin tags on toes); usually autosomal dominant inheritance, while group three encompasses scalp ACC with associated epidermal and organoid nevi. Group four includes ACC overlying embryologic malformations such as meningomyelocele, gastroschisis and omphalocele; while group five includes ACC with associated fetal papyraceous or placental infarcts. Group 6 consists of ACC that is associated with epidermolysis bullosa, while group 7 describes ACC localized to extremities without blistering, inheritance usually autosomal dominant or recessive. Group 8 is ACC caused by specific teratogens (methimazole, varicella and herpes simplex infection); while the last group [9] is ACC associated with malformation syndromes (trisomy 13; Adams Oliver syndrome; 4p-syndrome; many ectodermal dysplasia; Johanson-Blizzard syndrome; focal dermal hypoplasia; amniotic band disruption complex; XY gonadal dysgenesis e.t.c.). The cases in this report fit best into group one for case one as the baby did not have additional obvious malformations and the parents are cousins so the condition could have been inherited.

Case 2 best fits in group nine as baby had other malformations including a congenital heart disease, desquamating skin, hypoplastic finger and toe nails, visible dilated abdominal wall veins that could probably be described as Adams-Oliver Syndrome. The management of aplasia cutis congenita can be conservative or surgical depending on the presentation, size and location of defect. Conservative treatment includes the application of topical ointments such as silver sulfadiazine dressing, betadine, sofratulle dressing and use of antibiotics. Wound protection, infection prevention and nutrient supplementation are key to ensure positive treatment outcomes [13, 20]. These conservative methods are recommended if the defect is only at the skin level without damage to the bones and if the area of the defect is less than 3 cm [3, 7]. The conservative treatment exposes the patient to the risk of desiccation and necrosis of the aplasia cutis congenita tenuous wound bed and related morbidities, superior sagittal sinus thrombosis, hemorrhage, infection and slow wound healing [21]. In contrast, surgical intervention caries the risks of anesthesia, massive hemorrhage, scalp flap necrosis, skin graft loss, infection and donor site morbidity. Both cases presented in this report were managed conservatively with the plan of surgical intervention when infection was controlled and sufficient flaps could be raised.

Unfortunately none had surgery at the point of discharge although case 1 is still on follow up at the clinics. Surgical treatments include split-thickness or full-thickness skin grafts, acellular dermal matrices, autologous epithelial transplantation, tissue expansion, cranioplasty and so on. Conversely, skin grafts are necessary when the defected area is large and involves critical organs or tissues such as the scalp while also accompanied by the exposure of large blood vessels or sagittal sinus [7]. The timing of surgery remains controversial because of the risks involved with both conservative and surgical approaches [21]. Major complications of aplasia cutis congenita are rare, but can include haemorrhage, secondary local infection, meningitis, sagittal sinus thrombosis, bone affectation and death. Complications can also result from associated congenital malformations involving the cardiovascular, gastrointestinal, genitourinary and central nervous systems, when present [10]. The prognosis of aplasia cutis congenita is usually good, but if it is associated with other anomalies, the prognosis is dependent on the severity of the associated abnormalities [10]. Full-thickness defects of the scalp, skull and dura are associated with a mortality rate of greater than 50% [10].

## Conclusion

In conclusion, ACC although rare, poses great challenges to clinicians in sub-Saharan Africa when it occurs, especially when the defects are large or when it is associated with other congenital malformations. This, to the authors knowledge presents the first reported cases of ACC with scalp defects from West Africa.
